# Fatal Escherichia coli Necrotizing Fasciitis in a Patient With End-Stage Renal Disease and Type 2 Diabetes Mellitus

**DOI:** 10.7759/cureus.106141

**Published:** 2026-03-30

**Authors:** Caleb A Clark, Paige Boyd, Stephen Richbart, Imran Khawaja

**Affiliations:** 1 Internal Medicine, Marshall University Joan C. Edwards School of Medicine, Huntington, USA; 2 Medicine, Marshall University Joan C. Edwards School of Medicine, Huntington, USA; 3 Internal Medicine Residency Program, Marshall University Joan C. Edwards School of Medicine, Huntington, USA; 4 Pulmonary Medicine, Marshall University Joan C. Edwards School of Medicine, Huntington, USA

**Keywords:** case report, diabetes mellitus, end-stage renal disease, escherichia coli, necrotizing fasciitis, septic shock

## Abstract

Necrotizing fasciitis (NF) is a rare but life-threatening soft tissue infection characterized by rapid necrosis of fascia and surrounding tissues. While NF is most commonly associated with group A Streptococcus or polymicrobial etiologies, monomicrobial cases due to *Escherichia (E.) coli* are uncommon. When they do occur, these infections are often highly virulent and frequently fatal, especially in immunocompromised patients.

This report describes a fatal case of monomicrobial *E. coli* NF in a 64-year-old woman with end-stage renal disease (ESRD) on hemodialysis and type 2 diabetes mellitus. The infection originated from a urinary tract infection and vulvar abscess, culminating in septic shock and multiorgan failure. Blood and wound cultures grew *E. coli*. Despite aggressive resuscitation, broad-spectrum antibiotics, and emergent surgical debridement, the patient deteriorated rapidly and died within three days of admission.

This case highlights the aggressive nature of gram-negative monomicrobial NF in immunocompromised patients and underscores the importance of early recognition, prompt surgical intervention, and multidisciplinary critical care management.

## Introduction

Necrotizing fasciitis (NF) is a rapidly progressive soft tissue infection associated with significant morbidity and mortality. Mortality rates remain high despite advances in critical care and surgical management, with reported mortality ranging from 20% to over 50% in severe cases [1,2]. NF is traditionally classified into type I (polymicrobial) and type II (monomicrobial, most commonly group A Streptococcus) infections [1]. Increasing recognition of monomicrobial gram-negative NF, including cases caused by *Escherichia coli* (*E. coli*), has been reported in immunocompromised hosts [3,4].

Monomicrobial *E. coli* NF is uncommon but carries a high mortality rate, particularly in patients with diabetes mellitus, chronic kidney disease, malignancy, or recent surgical interventions [5]. Early diagnosis is challenging, as initial cutaneous findings may be subtle despite profound systemic toxicity. Delays in surgical debridement significantly worsen outcomes and are strongly associated with increased mortality [6].

We present a fatal case of monomicrobial *E. coli* NF in a patient with multiple comorbidities, emphasizing clinical presentation, management challenges, and relevant pathophysiology.

## Case presentation

A 64-year-old woman with end-stage renal disease (ESRD) on hemodialysis, type 2 diabetes mellitus, and hepatitis C virus (HCV) infection presented with hypotension, fever, and altered mental status. Initial vital signs revealed a blood pressure of 75/42 mmHg, heart rate greater than 110 beats per minute, temperature of 38.5 °C, and an arterial blood gas (ABG) demonstrating a pH of 7.50, pCO₂ of 29 mmHg, and pO₂ of 67 mmHg, consistent with a primary respiratory alkalosis with concurrent hypoxemia. She was admitted to the intensive care unit (ICU) for septic shock.

Initial resuscitation included 1.75 L (30 mL/kg) of lactated Ringer’s solution and initiation of norepinephrine, later requiring the addition of vasopressin. Empiric antibiotics with vancomycin and cefepime were administered after cultures were obtained.

Admission laboratory studies revealed significant abnormalities reflecting the severity of infection and underlying comorbidities (Table 1). Complete blood count (CBC) demonstrated a white blood cell (WBC) count of 7.46 ×10³/µL with lymphopenia (absolute lymphocyte count 0.37 ×10³/µL), hemoglobin of 7.7 g/dL, and platelet count of 159 ×10³/µL. A comprehensive metabolic panel (CMP) was remarkable for hyponatremia (sodium 123 mEq/L), hypokalemia (potassium 2.7 mEq/L), hypochloremia (chloride 88 mEq/L), acute-on-chronic kidney injury (creatinine 4.55 mg/dL, up from a baseline of 2.7 mg/dL), hypocalcemia (calcium 7.2 mg/dL), and hypoalbuminemia (albumin 2.4 g/dL). Inflammatory markers were markedly elevated, with a C-reactive protein (CRP) of 31.8 mg/dL and procalcitonin greater than 100 ng/mL. Lactic acid on admission was 3.2 mmol/L and trended upward to 3.9 and subsequently 4.3 mmol/L despite fluid resuscitation. Coagulation studies were deranged with an international normalized ratio (INR) of 2.4, prothrombin time (PT) of 28 seconds, and activated partial thromboplastin time (aPTT) of 37 seconds, consistent with disseminated intravascular coagulation (DIC) in the clinical context. B-type natriuretic peptide (BNP) was elevated at 1,018 pg/mL, while serial troponin levels remained stably elevated at 41, 37, and 39 ng/L at 0, 2, and 4 hours, respectively, likely reflecting type 2 myocardial injury in the setting of demand ischemia. Urinalysis (UA) showed a large amount of leukocyte esterase with negative nitrites. Hepatitis C antibody was positive. The Laboratory Risk Indicator for Necrotizing Fasciitis (LRINEC) score was calculated at 10, representing a 93.4% positive predictive value for necrotizing soft tissue infection [7].

**Table 1:** Admission laboratory values PT: prothrombin time; aPTT: activated partial thromboplastin time; BNP: B-type natriuretic peptide; ABG: arterial blood gas; LRINEC: Laboratory Risk Indicator for Necrotizing Fasciitis

Laboratory Parameter	Admission Value	Reference Range
White blood cell count (×10³/µL)	7.46	4.5–11.0
Hemoglobin (g/dL)	7.7	12.0–16.0
Platelet count (×10³/µL)	159	150–400
Absolute lymphocyte count (×10³/µL)	0.37	1.0–4.8
Absolute neutrophil count (×10³/µL)	5.45	1.8–7.7
Sodium (mEq/L)	123	136–145
Potassium (mEq/L)	2.7	3.5–5.0
Chloride (mEq/L)	88	98–106
Creatinine (mg/dL)	4.55 (baseline 2.7)	0.6–1.2
Calcium (mg/dL)	7.2	8.5–10.5
Albumin (g/dL)	2.4	3.5–5.0
Glucose (mg/dL)	104	70–100
Serum osmolality (mOsm/kg)	279	275–295
C-reactive protein (mg/dL)	31.8	<0.5
Procalcitonin (ng/mL)	>100	<0.1
Lactic acid (mmol/L)	3.2 → 3.9 → 4.3	0.5–2.0
INR	2.4	0.8–1.2
PT (seconds)	28	11–13.5
aPTT (seconds)	37	25–35
BNP (pg/mL)	1,018	<100
Troponin (ng/L)	41 (0h), 37 (2h), 39 (4h)	<14
ABG pH / pCO₂ / pO₂	7.50 / 29 / 67	7.35–7.45 / 35–45 / 80–100
LRINEC score	10	≥6: suspicious; ≥8: strongly predictive

A repeat CBC obtained approximately 12 hours after admission demonstrated an interval decline in WBC to 3.15 ×10³/µL with hemoglobin of 8.8 g/dL and platelets of 149 ×10³/µL (Table 2). The development of leukopenia in the setting of worsening septic shock suggested bone marrow suppression and was considered a poor prognostic indicator.

**Table 2:** Serial complete blood count values

Parameter	Admission	12 Hours Post-Admission
WBC (×10³/µL)	7.46	3.15
Hemoglobin (g/dL)	7.7	8.8
Platelets (×10³/µL)	159	149

Physical examination revealed a stage III sacral pressure ulcer. During Foley catheter placement, a vulvar abscess with purulent drainage was discovered.

**Figure 1:**
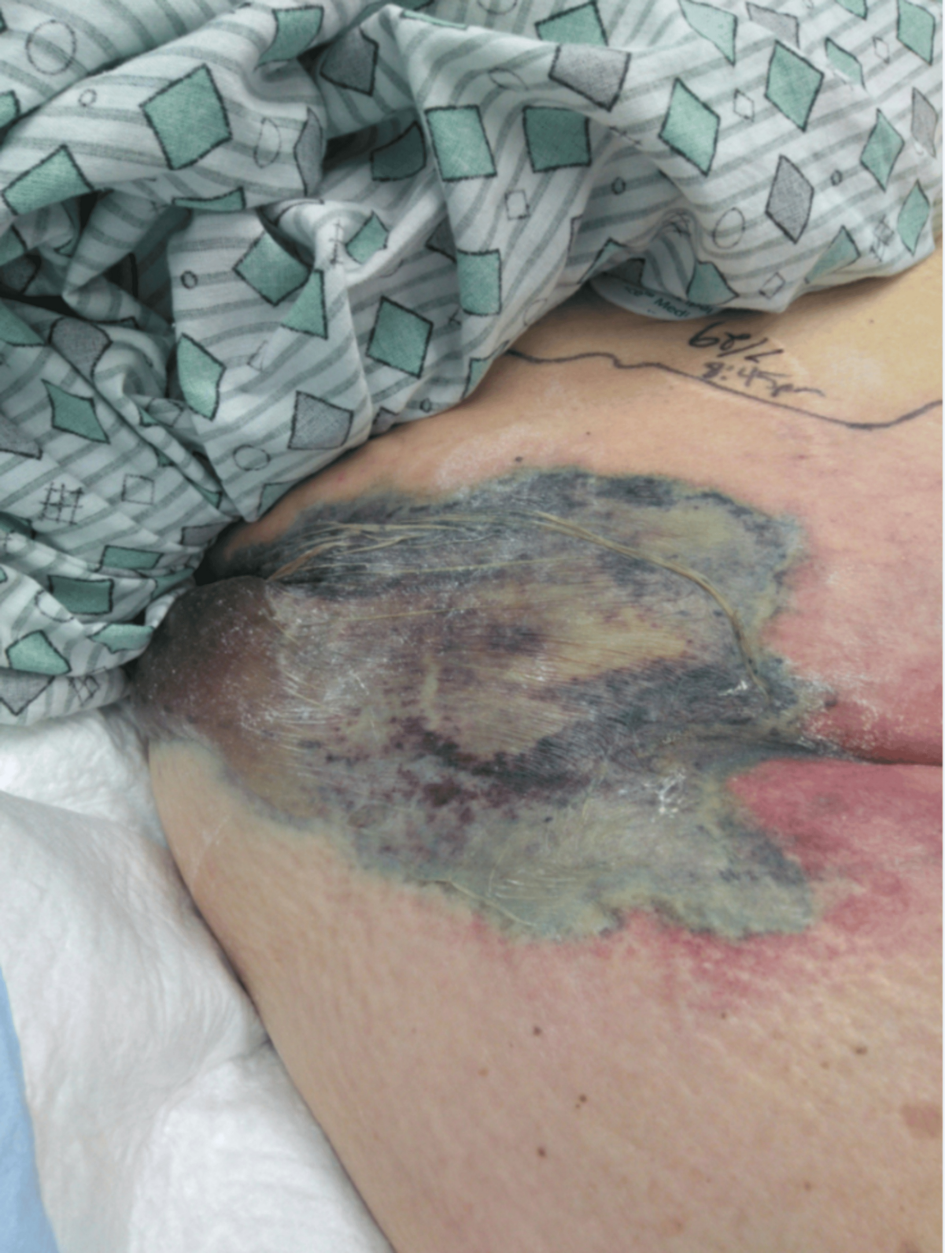
Cutaneous findings of necrotizing fasciitis involving the right buttock and perineum Early skin manifestations demonstrate violaceous discoloration, ecchymosis, and evolving bullous changes along the gluteal fold. The outlined markings indicate the clinically suspected borders of infection used to monitor progression prior to surgical exploration.

**Figure 2:**
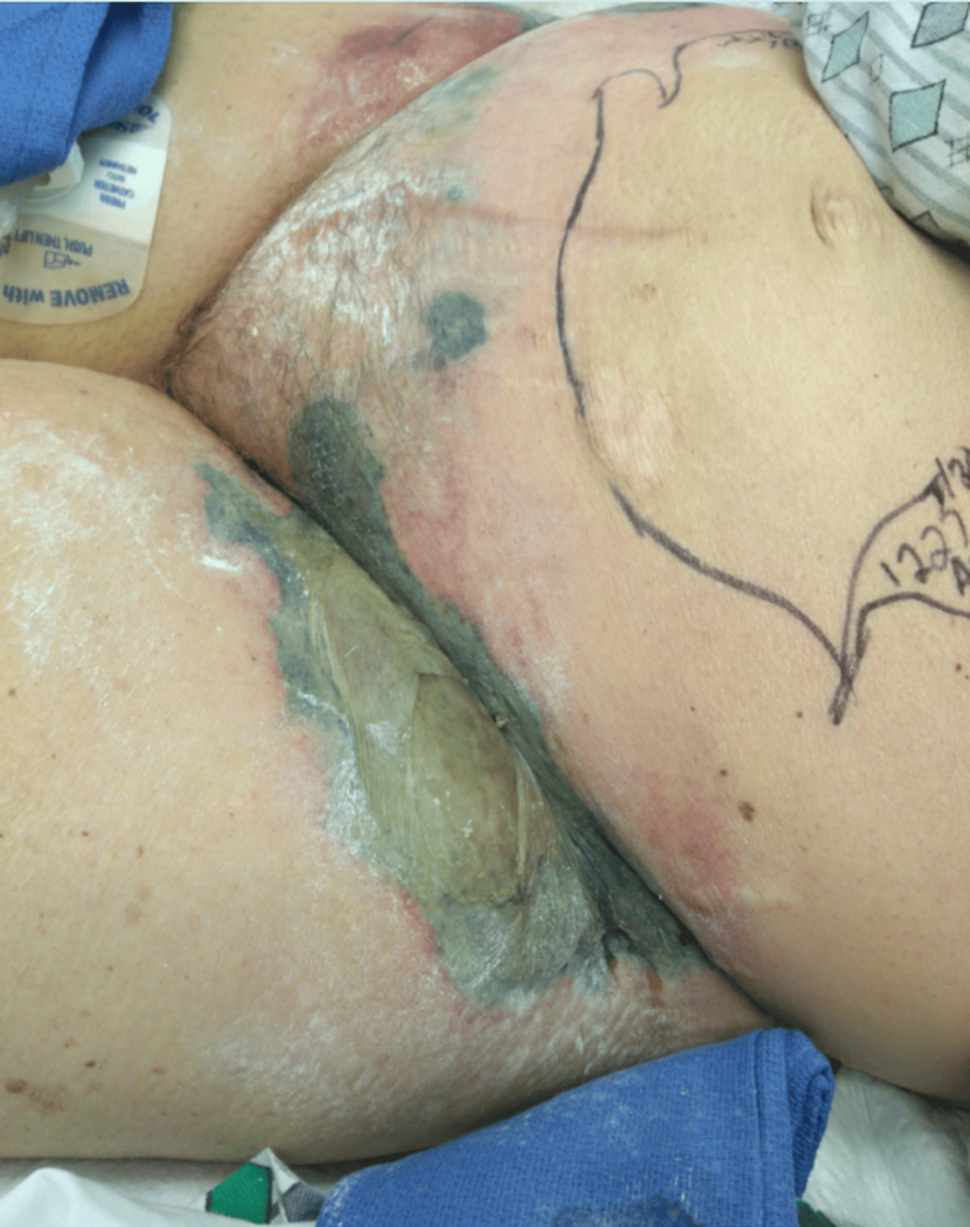
Progressive cutaneous necrosis of the gluteal and perineal region Extensive skin discoloration with hemorrhagic bullae and evolving tissue necrosis involving the gluteal fold and perineal region. These findings are consistent with advanced necrotizing soft tissue infection and illustrate the rapid progression of cutaneous involvement prior to surgical exploration.

External examination demonstrated rapidly progressive skin discoloration, ecchymosis, and bullous changes involving the right buttock and perineal region, concerning for necrotizing soft tissue infection (NSTI) (Figure 1 and Figure 2).

Given concern for necrotizing infection of the perineum, surgical consultation was obtained, and the patient underwent emergent operative exploration. Intraoperative findings confirmed extensive necrosis involving the right buttock, right labia, and perineum, consistent with NF. Surgical debridement was performed, and antimicrobial therapy was escalated to meropenem and clindamycin in addition to vancomycin. Despite initial debridement, the infection progressed with the development of subcutaneous crepitus and bullae extending toward the anterior abdominal wall. Blood cultures and wound cultures grew *E.coli*. Urine cultures grew *Enterococcus faecalis*.

Given rapid deterioration, refractory septic shock, and poor overall prognosis in the context of multiple comorbidities, the family declined additional extensive debridement. The patient continued to worsen and died three days after admission due to refractory septic shock and multiorgan failure.

## Discussion

NF caused by gram-negative organisms represents a particularly aggressive subset of soft tissue infections. While most cases are polymicrobial or caused by group A Streptococcus, monomicrobial infections due to *E. coli *have been increasingly reported in immunocompromised hosts [3,4]. These infections are frequently associated with significant systemic toxicity and rapid clinical deterioration.

Several host factors increase susceptibility to severe soft tissue infections, particularly diabetes mellitus, chronic kidney disease, malignancy, and immunosuppression [5,7,8]. Patients with diabetes demonstrate impaired neutrophil chemotaxis and phagocytosis, while ESRD is associated with immune dysfunction and impaired wound healing. Microvascular disease further contributes to tissue hypoperfusion, creating an environment favorable for bacterial proliferation and toxin-mediated tissue destruction [8,9].

*E. coli *NF is believed to arise through several mechanisms. In many cases, infection originates from gastrointestinal or genitourinary sources, with bacterial translocation into adjacent soft tissues or hematogenous spread [3]. Virulent strains of *E. coli*, particularly extraintestinal pathogenic* E. coli* (ExPEC), possess virulence factors, including adhesins, cytotoxins, and iron-acquisition systems that facilitate rapid invasion of soft tissues [4]. These organisms can trigger a severe inflammatory cascade leading to septic shock and multiorgan dysfunction.

The laboratory findings in this case illustrate the severity of systemic involvement characteristic of monomicrobial gram-negative NF. The markedly elevated procalcitonin (>100 ng/mL) and CRP (31.8 mg/dL) reflect the intensity of the inflammatory response. Elevated lactic acid that trended upward despite resuscitation (3.2 to 4.3 mmol/L) indicated persistent tissue hypoperfusion and was a harbinger of poor prognosis. The coagulopathy (INR 2.4, PT 28 seconds) in combination with thrombocytopenia suggested evolving DIC, a recognized complication of severe sepsis. The LRINEC score of 10 in this patient corresponded to a 93.4% positive predictive value for NSTI and supported the clinical suspicion prior to surgical confirmation [10]. Prior case series of monomicrobial *E. coli* NF have similarly reported profound inflammatory marker elevation and high rates of coagulopathy, with mortality exceeding 50% in patients presenting with septic shock [4,7,8].

Early diagnosis of NF remains challenging because initial cutaneous findings may be minimal despite extensive deep tissue involvement [2]. Clinical features that should raise suspicion include severe pain out of proportion to physical findings, rapidly progressing erythema or edema, crepitus, bullae formation, and systemic toxicity [1]. Imaging studies such as computed tomography (CT) or magnetic resonance imaging (MRI) may demonstrate fascial thickening or subcutaneous gas, but definitive diagnosis often requires surgical exploration.

Management requires a multidisciplinary approach centered on prompt surgical debridement, aggressive resuscitation, and broad-spectrum antimicrobial therapy [7]. Empiric antibiotic regimens typically include agents targeting gram-positive, gram-negative, and anaerobic organisms until culture data become available. Clindamycin is frequently included due to its ability to suppress toxin production in toxin-mediated infections [1]. Repeated surgical debridement is often necessary to control disease progression.

Despite aggressive management, mortality rates for NF remain high, particularly among patients presenting with septic shock or multiple comorbidities [1,2]. Mortality rates exceeding 50% have been reported in patients with monomicrobial gram-negative NF, particularly infections caused by virulent *E. coli *strains [4]. Early recognition and immediate surgical intervention remain the most important determinants of survival, with multiple studies demonstrating significantly increased mortality when debridement is delayed [6].

This case highlights the fulminant course that monomicrobial *E. coli *NF can take in patients with significant comorbid conditions. Even with prompt recognition and guideline-directed therapy, disease progression may be rapid and difficult to control.

## Conclusions

Monomicrobial *Escherichia coli *necrotizing fasciitis is rare but highly lethal, particularly in immunocompromised patients. Early suspicion and immediate surgical intervention are critical for survival. Clinicians should maintain heightened vigilance for necrotizing infection in patients with septic shock and subtle perineal or soft tissue findings.

Further research is needed to better characterize virulence mechanisms and identify potential adjunctive therapeutic strategies.
